# Neighborhood Child Opportunity and Preterm Birth Rates by Race and Ethnicity

**DOI:** 10.1001/jamanetworkopen.2024.32766

**Published:** 2024-09-11

**Authors:** Candice Belanoff, Adriana Black, Collette N. Ncube, Dolores Acevedo-Garcia, Joanna Almeida

**Affiliations:** 1 Department of Community Health Sciences, Boston University School of Public Health, Boston, Massachusetts; 2 Office of the Vice Chancellor for Health Affairs, University of Illinois, Chicago; 3 Department of Epidemiology, Boston University School of Public Health, Boston, Massachusetts; 4 Institute for Child, Youth & Family Policy, Brandeis University, Waltham, Massachusetts; 5 Simmons University School of Social Work, Boston, Massachusetts

## Abstract

**Importance:**

In the US, infants born to non-Hispanic Black birthing parents are 50% more likely to be born preterm than those born to non-Hispanic White birthing parents, and individual-level factors do not fully account for this inequity. Neighborhood context, rooted in historic patterns of structural racism, may facilitate understanding patterns of inequity in preterm birth.

**Objective:**

To estimate the association between neighborhood opportunity level, measured by the Child Opportunity Index (COI), and preterm birth among infants in Massachusetts.

**Design, Setting, and Participants:**

In this cross-sectional, population-based study, Massachusetts birth certificates from 3 large metropolitan areas (Boston, Springfield, and Worcester) were linked to US Census tract–level data from the COI, and log binomial regression models and generalized estimating equations were fit to examine associations of different levels of opportunity with preterm birth. Singleton infants born in Massachusetts between February 1, 2011, and December 31, 2015, were included. Analyses were originally conducted in 2019 and updated in 2024.

**Exposure:**

Level of child opportunity (measured by the COI) at the US Census tract level. Race and ethnicity were ascertained from the birth certificate, as reported by the birthing parent.

**Main Outcomes and Measures:**

Live birth before 37 completed weeks’ gestation.

**Results:**

The analytic dataset included 267 553 infants, of whom 18.9% were born to Hispanic, 10.1% to non-Hispanic Asian or Pacific Islander, 10.1% to non-Hispanic Black, and 61.0% to non-Hispanic White birthing parents. More than half of infants born to non-Hispanic Black and Hispanic birthing parents were born into very low opportunity neighborhoods, and in crude models, this was associated with greater prevalence of preterm birth relative to very high opportunity neighborhoods (prevalence ratio, 1.44; 95% CI, 1.37-1.52). After adjustment for covariates, infants born into very low opportunity neighborhoods still had a greater prevalence of preterm birth (prevalence ratio, 1.16; 95% CI, 1.10-1.23).

**Conclusions and Relevance:**

In this cross-sectional study of neighborhood opportunity and preterm birth, elevated risk associated with exposure to a very low opportunity neighborhood, coupled with the disproportionate exposure by race and ethnicity, points to a modifiable factor that may contribute to racial and ethnic inequities in preterm birth. Future research should investigate interventions that seek to address neighborhood opportunity.

## Key Points

**Question:**

Is level of neighborhood child opportunity, a marker of structural racism, associated with preterm birth in Massachusetts?

**Findings:**

In this cross-sectional study linking Massachusetts birth certificates to the neighborhood-level Child Opportunity Index, among 267 553 infants, those born to non-Hispanic Black as well as Hispanic birthing parents were far more likely to be born into very low opportunity areas than other infants. Additionally, living in a very low vs very high opportunity area was associated with 16% higher risk of preterm birth.

**Meaning:**

These findings suggest that very low neighborhood social opportunity may pose a risk for preterm birth above and beyond individual factors and, as such, contribute to the racial gap in preterm birth in Massachusetts.

## Teaser

This cross-sectional study of infants born in Massachusetts examines whether a multidimensional measure of neighborhood child opportunity is associated with preterm birth.

## Introduction

Preterm birth (PTB) is 1 of 2 leading causes of infant mortality overall in the US and the most common cause among infants born to non-Hispanic Black (hereafter referred to as Black) birthing parents.^1^ Among infants who survive, PTB negatively impacts physical, behavioral, and mental health across the life course.^2^ In 2022 in the US, 14.6% of infants born to Black and 10.1% of infants born to Hispanic birthing parents were born preterm (<37 completed weeks’ gestation) compared with 9.4% born to non-Hispanic White (hereafter referred to as White) birthing parents.^3^ The specific causes of PTB and mechanisms driving racial and ethnic inequities are incompletely understood,^4^ and prevention and equity efforts therefore remain challenging.^2^ Known individual factors associated with PTB, including sociodemographic (eg, educational level, marital status, and age), behavioral or experiential (eg, plurality, substance use, and intimate partner violence),^2^ and medical (eg, infection and gestational diabetes),^4^ explain only some of the racial and ethnic gap.

Because of the limited capacity of individual-level factors to explain inequities, neighborhood social context has increasingly become a focus of PTB research,^5,6^ yielding consistent associations above and beyond individual-level factors.^7^ In a 2016 meta-analysis, people living in the most disadvantaged neighborhoods had a nearly 30% higher risk of delivering preterm compared with those living in the most advantaged neighborhoods.^5^ The disproportionate distribution of people by race and ethnicity into areas characterized by more or less opportunity and resources, a form of structural racism, can be directly linked to historic and current residential segregation and community disinvestment policies and practices.^8,9,10,11^ Racial residential segregation results in unfair differences in socioeconomic status and mobility^12^ and inequitable exposure to noxious physical and social environments. In addition, racial residential segregation is a fundamental cause of inequities in health.^13^ Results of a systematic review and meta-analysis found that residential segregation was associated with an increased risk of PTB among Black but not White birthing people,^10^ suggesting that residential segregation is harmless or possibly even advantageous to privileged racial groups. Krieger et al^9^ identified an independent association of historic redlining with contemporary PTB rates, even after accounting for individual determinants and contemporary census tract poverty level. Most of the existing research on neighborhoods and health has focused on a single aspect of neighborhood context (eg, poverty, violence, or segregation).^14^ Multidimensional measures of neighborhood opportunity capture the complexity of community life and may help illuminate both positive and negative factors, across a range of domains, associated with the health of residents.^14^

The growth of non-White populations in the US^15^ and the persistent segregation, particularly of Black and Hispanic groups, into lower opportunity areas^16^ increase the need to understand the role of neighborhood opportunity in PTB. As such, the overarching aim of this study was to understand the association between the neighborhood-level Child Opportunity Index 1.0 (COI), a multifaceted, composite measure of educational, health and environmental, and socioeconomic opportunity,^17^ and PTB among infants in Massachusetts. The COI has been shown to be associated with child mortality; pediatric health care use; child health markers, such as cortisol; and child health problems, such as injuries.^14,18,19^ Our research questions were (1) to what extent neighborhood-level child opportunity is associated with PTB above and beyond individual-level birthing parent characteristics and (2) whether differential exposure to neighborhood-level child opportunity contributes to racial inequities in PTB.

## Methods

### Procedure and Population

We linked data from the Massachusetts birth certificate data file for births occurring from February 1, 2011, to December 31, 2015, to neighborhood-level child opportunity data from the COI using 2010 US Census tract codes corresponding to the residential address of the birthing parent at the time of delivery. The study population included live-born, singleton births in Massachusetts to parents living within any of the 3 major metropolitan areas of Massachusetts (Boston, Worcester, or Springfield) at the time of delivery because the COI covered only the 100 largest metropolitan areas in the US.^17^ Infants born to people living in Massachusetts Census tracts outside the 3 COI-defined metropolitan areas or that were not geocoded were not included. Additionally, we used a complete case approach to constructing the analytic data file, excluding observations with missing data on the outcome of gestational age (n = 357 [0.13%]) or on any covariates (accounting for 3.8% of the original dataset). Most excluded cases lacked body mass index (BMI) (n = 6902) and educational attainment (n = 4536) data. The eTable in Supplement 1 shows a comparison of the complete-case dataset to the original dataset. Analyses were originally conducted in 2019 and updated in 2024. To maintain confidentiality, the Massachusetts Department of Public Health replaced US Census tract codes with unique identifiers before our analyses. Our final analytic sample of 267 553 singleton births represented 78.8% of all Massachusetts singleton live births^20^ during the study period. This study was approved by the institutional review boards of the Boston University Medical Campus and Massachusetts Department of Public Health. The Massachusetts Department of Public Health (MDPH) Confidential Data Officer granted the researchers access to a deidentified set of data generated from the state’s birth registry. No consent of individuals represented in the data was sought by the researchers. The study followed the Strengthening the Reporting of Observational Studies in Epidemiology (STROBE) reporting guideline.^21^

### Measures

The outcome of interest, PTB, was defined as any live birth occurring before 37 completed weeks’ gestation and was measured using the obstetric estimate on the birth certificate. The COI^17^ is a US Census tract–level (proxy for neighborhoods) composite measure of resources and conditions associated with child health and development. Neighborhood opportunity is conceptualized in 3 domains with corresponding subindexes: educational opportunities (eg, educational attainment and student reading proficiency levels), health and environmental opportunities (eg, proximity to health care facilities and toxic waste sites), and social and economic opportunities (eg, poverty rate and proximity to employment). The composite COI score for each census tract uses the metropolitan area mean score across the 3 subindexes, categorized into quintiles (very low, low, moderate, high, and very high) and normed to the metropolitan area. The COI includes measures captured during and around the year 2010, establishing extant conditions for our sample born between 2011 and 2015.

Individual-level covariates were derived from birth certificates and included birthing parent race and ethnicity (non-Hispanic Asian or Pacific Islander [hereafter referred to as Asian or Pacific Islander], Black, Hispanic, and White). We use the term *Hispanic* to be consistent with language on the birth certificate. Although *mother* and *maternal* are used on the birth certificate, we use the term *birthing parent* to be inclusive of people who do not identify as female. Additionally, we controlled for birthing parent age, educational level, marital status, nativity, private insurance, any perinatal health conditions, pre-pregnancy BMI, and metropolitan area of residence (Boston, Worcester, or Springfield).

### Statistical Analysis

We first conducted analyses describing the frequency and percentage distributions of the sample. We then conducted bivariate analyses to examine associations between COI and race and ethnicity and between PTB and all variables using χ^2^ statistics for tests of significance. To investigate the potential contribution of COI to racial inequities in PTB, we conducted a series of log binomial regression models, which included the following: (1) race and ethnicity alone, (2) COI alone, (3) race and ethnicity and COI together, and (4) a full model including race and ethnicity, COI, and all covariates. For all regression models, we used generalized estimating equations to account for clustering at the US Census tract level. We considered a 2-sided *P* < .05 or a 95% CI not containing 1.0 to be statistically significant.

Interpretation of our findings was aided by criteria for the assessment of racial disparities from Ward et al,^22^ who direct researchers to address the following 3 questions about the exposure and outcome: (1) “Is there a difference in the prevalence of the outcome between groups?” (2) “Is there a difference in the prevalence of the exposure between groups?” and (3) “Does the relationship between the exposure and outcome differ between groups?” Our bivariate analyses addressed the first 2 questions, and to address question 3, we tested an interaction between race and ethnicity and COI in the context of our full model. All statistical analyses were conducted using SAS software, version 9.4 (SAS Institute Inc).^23^

## Results

### Descriptive Analyses

The analytic dataset included 267 553 infants. Approximately one-quarter (24.8%) of all infants in the sample were born into very low opportunity neighborhoods and between 18.3% and 19.8% of infants were born into neighborhoods characterized as low, moderate, high, and very high opportunity (Table 1). The racial and ethnic distribution of our study sample closely mirrored that of the overall population of people who gave birth in Massachusetts during the study period,^24^ with 10.1% Asian or Pacific Islander, 10.1% Black, 18.9% Hispanic, and 61.0% White. The largest proportions of birthing parents resided in the Boston metropolitan area (74.4%), were between the ages of 26 and 34 years (55.8%), were married (67.0%), were born in the US (67.7%), had at least a 4-year college degree (48.7%), had some perinatal health condition noted (58.2%), and had a pre-pregnancy BMI between 18.5 and 24.9 (calculated as weight in kilograms divided by height in meters squared) (49.6%). The percentage of PTB was highest among infants born to Black birthing parents (8.4%), followed by Hispanic (7.3%), Asian or Pacific Islander (5.8%), and White (5.8%) (Table 1.)

**Table 1.** Sample Description and Prevalence of PTB Among Birthing Parents in the Metropolitan Areas of Boston, Worcester, and Springfield, Massachusetts, February 2011 to December 2015

Characteristic	No. (%) of birthing parents	PTB rate, No. (%)
Race and ethnicity		
Hispanic	50 559 (18.9)	3672 (7.3)
Non-Hispanic Asian or Pacific Islander	26 948 (10.1)	1565 (5.8)
Non-Hispanic Black	26 911 (10.1)	2252 (8.4)
Non-Hispanic White	163 135 (61.0)	9377 (5.8)
Child Opportunity Index level		
Very high	50 333 (18.8)	2633 (5.2)
High	49 030 (18.3)	2844 (5.8)
Moderate	52 907 (19.8)	3234 (6.1)
Low	49 004 (18.3)	3169 (6.5)
Very low	66 279 (24.8)	4986 (7.5)
Metropolitan area of residence		
Boston	198 992 (74.4)	12 257 (6.2)
Springfield	30 954 (11.6)	2032 (6.6)
Worcester	37 607 (14.1)	2577 (6.9)
Age range, y		
12-19	10 152 (3.8)	805 (7.9)
20-25	46 363 (17.3)	3029 (6.5)
26-34	149 244 (55.8)	8638 (5.8)
35-39	50 067 (18.7)	3348 (6.7)
40-44	10 969 (4.1)	968 (8.8)
≥45	758 (0.3)	78 (10.3)
Marital status		
Divorced	5659 (2.1)	456 (8.1)
Married	179 254 (67.0)	10 092 (5.6)
Never married	82 428 (30.8)	6296 (7.6)
Other relationship	212 (0.1)	22 (10.4)
Nativity		
US born	181 089 (67.7)	11 362 (6.3)
Non–US born	86 464 (32.3)	5504 (6.4)
Educational level		
Less than high school	26 499 (9.9)	2028 (7.7)
High school	45 050 (16.8)	3411 (7.6)
Some college	65 720 (24.6)	4502 (6.9)
4-y degree	67 848 (25.4)	3759 (5.5)
Graduate school	62 436 (23.3)	3166 (5.1)
Private insurance for delivery		
Yes	206 709 (77.3)	12 555 (6.1)
No	60 844 (22.7)	4311 (7.1)
Any perinatal health conditions^a^		
No	111 895 (41.8)	3814 (3.4)
Yes	155 658 (58.2)	13 052 (8.4)
Pre-pregnancy BMI		
<18.5	7541 (2.8)	566 (7.5)
18.5-24.9	132 738 (49.6)	7739 (5.8)
25.0-29.9	72 866 (27.2)	4516 (6.2)
30.0-34.9	30 764 (11.5)	2152 (7.0)
35.0-39.9	14 253 (5.3)	1097 (7.7)
≥40	9391 (3.5)	796 (8.5)

Abbreviations: BMI, body mass index (calculated as weight in kilograms divided by height in meters squared); PTB, preterm birth.

^a^ Includes acute and chronic lung disease, anemia, cardiac disease, prediabetes, pre-pregnancy diabetes, gestational diabetes, hemoglobinopathy non–sickle cell anemia, sickle cell anemia, hydramnios, hypercoagulable conditions, hypertension pre-pregnancy, hypertension preeclampsia, hypertension eclampsia, hypertension gestational, incompetent cervix, lupus erythematosus, maternal cancers diagnosed during pregnancy, maternal phenylketonuria, oligohydramnios, preterm labor before current pregnancy, previous infant with birth defects, previous infant weighing 4000 g or more, previous preterm birth, previous cesarean delivery, other previous poor pregnancy outcome, renal disease, Rh sensitization, seizure disorders, vaginal bleeding, and weight gain or loss inappropriate for mother.

Race and ethnicity were closely associated with the level of neighborhood opportunity into which infants were born. Infants whose birthing parents were Black or Hispanic were more likely to be born into very low child opportunity neighborhoods (54.5% and 54.3%, respectively) than were infants born to White and Asian or Pacific Islander parents (11.8% and 19.6%, respectively) (Figure). Infants born to Black and Hispanic birthing parents were also least likely to be born into very high opportunity neighborhoods (6.7% and 6.0%, respectively) compared with 23.4% and 26.0% of infants born to White and Asian or Pacific Islander birthing parents, respectively (*P* < .001).

**Figure.**
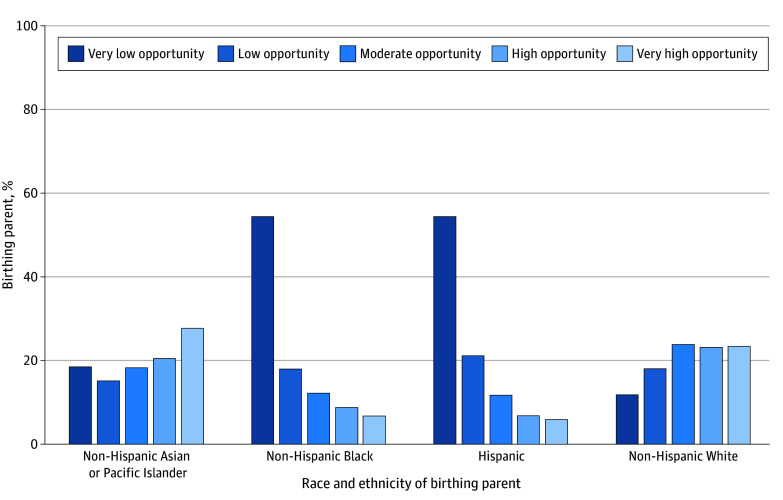
Race and Ethnicity of Birthing Parents by Neighborhood Opportunity Level

### Regression Models

#### Race and Ethnicity and PTB

In the unadjusted model of race and ethnicity and PTB, infants born to Black birthing parents had a 42% higher prevalence of PTB (prevalence ratio [PR], 1.42; 95% CI, 1.35-1.49), and those born to Hispanic birthing parents had a 24% higher prevalence of PTB (PR, 1.24; 95% CI, 1.20-1.29) compared with those born to White birthing parents (Table 2). Infants born to Asian or Pacific Islander birthing parents did not significantly differ from those born to White birthing parents in their risk of PTB (Table 2).

**Table 2.** Level of Child Opportunity and Prevalence of Preterm Birth in the Metropolitan Areas of Boston, Worcester, and Springfield, Massachusetts, February 2011- December 2015

Characteristic	Prevalence ratio (95% CI)			
	Race and ethnicity only	COI only	Race and ethnicity and COI	Full model^a^
Race and ethnicity				
Hispanic	1.24 (1.20-1.29)	NA	1.17 (1.12-1.22)	1.13 (1.08-1.18)
Non-Hispanic Asian/Pacific Islander	1.02 (0.96-1.07)	NA	1.01 (0.96-1.07)	1.15 (1.08-1.22)
Non-Hispanic Black	1.42 (1.35-1.49)	NA	1.34 (1.27-1.41)	1.28 (1.21-1.35)
Non-Hispanic White	1.0 [Reference]	NA	1.0 [Reference]	1.0 [Reference]
COI level				
Very high	NA	1.0 [Reference]	1.0 [Reference]	1.0 [Reference]
High	NA	1.11 (1.05-1.18)	1.10 (1.04-1.17)	1.08 (1.02-1.15)
Moderate	NA	1.17 (1.10-1.24)	1.15 (1.09-1.22)	1.11 (1.05-1.17)
Low	NA	1.24 (1.17-1.31)	1.18 (1.12-1.25)	1.11 (1.05-1.17)
Very low	NA	1.44 (1.37-1.52)	1.29 (1.22-1.36)	1.16 (1.10-1.23)
Metropolitan area of residence)				
Boston	NA	NA	NA	1.0 [Reference]
Springfield	NA	NA	NA	0.87 (0.83-0.92)
Worcester	NA	NA	NA	1.07 (1.03-1.12)
Age range, y				
12-19	NA	NA	NA	1.01 (0.94-1.08)
20-25	NA	NA	NA	0.91 (0.88-0.96)
26-34	NA	NA	NA	1.0 [Reference]
35-39	NA	NA	NA	1.12 (1.08-1.16)
40-44	NA	NA	NA	1.38 (1.29-1.47)
≥45	NA	NA	NA	1.53 (1.24-1.89)
Marital status				
Married	NA	NA	NA	1.0 [Reference]
Divorced	NA	NA	NA	1.17 (1.07-1.29)
Never married	NA	NA	NA	1.17 (1.13-1.22)
Other relationship	NA	NA	NA	1.39 (0.92-2.10)
Nativity				
US born	NA	NA	NA	1.0 [Reference]
Non–US born	NA	NA	NA	0.91 (0.87-0.95)
Educational level				
Less than high school	NA	NA	NA	1.15 (1.08-1.23)
High school	NA	NA	NA	1.18 (1.12-1.24)
Some college	NA	NA	NA	1.09 (1.04-1.14)
4-y degree	NA	NA	NA	1.0 [Reference]
Graduate school	NA	NA	NA	0.94 (0.89-0.98)
Insurance for delivery				
Private	NA	NA	NA	1.0 [Reference]
Nonprivate	NA	NA	NA	0.98 (0.95-1.02)
Any perinatal health conditions				
Any	NA	NA	NA	2.37 (2.27-2.48)
None	NA	NA	NA	1.0 [Reference]
Pre-pregnancy BMI				
<18.5	NA	NA	NA	1.25 (1.16-1.36)
18.5-24.9	NA	NA	NA	1.0 [Reference]
25.0-29.9	NA	NA	NA	0.96 (0.93-1.00)
30.0-34.9	NA	NA	NA	0.99 (0.95-1.05)
35.0-39.9	NA	NA	NA	1.04 (0.97-1.11)
≥40	NA	NA	NA	1.07 (0.99-1.15)

Abbreviations: BMI, body mass index (calculated as weight in kilograms divided by height in meters squared); COI, Child Opportunity Index; NA, not applicable.

^a^ In addition to race and ethnicity, the full model adjusts for birthing parent age, marital status, educational level, private insurer, any perinatal health issues (including acute and chronic lung disease, anemia, cardiac disease, prediabetes, pre-pregnancy diabetes, gestational diabetes, hemoglobinopathy non–sickle cell anemia, sickle cell anemia, hydramnios, hypercoagulable conditions, hypertension pre-pregnancy, hypertension preeclampsia, hypertension eclampsia, hypertension gestational, incompetent cervix, lupus erythematosus, maternal cancers diagnosed during pregnancy, maternal phenylketonuria, oligohydramnios, preterm labor before this pregnancy, previous infant with birth defects, previous infant weighing 4000 g or more, previous preterm birth, previous cesarean delivery, other previous poor pregnancy outcome, renal disease, Rh sensitization, seizure disorders, vaginal bleeding, and weight gain or loss inappropriate for mother), and BMI.

#### COI and PTB

We observed a graded association between neighborhood opportunity level and risk of PTB with decreasing opportunity associated with increased risk of PTB (Table 2). Compared with the very high opportunity group, infants born into very low opportunity areas had a 44% higher prevalence of PTB (PR, 1.44; 95% CI, 1.37-1.52).

#### COI and Race and Ethnicity

In a model with both race and ethnicity and COI, the association between each and PTB was somewhat attenuated (Table 2). The risk of PTB for infants born to Black birthing parents was reduced (PR, 1.34; 95% CI, 1.27-1.41), as was the risk associated with living in a very low’ opportunity area (PR, 1.29; 95% CI, 1.22-1.36).

### Fully Adjusted Model

In the fully adjusted model, the association of the COI with risk of PTB was further attenuated; however, infants born into very low opportunity areas still had 16% higher prevalence of PTB than those born into very high opportunity areas (PR, 1.16; 95% CI, 1.10-1.23) (Table 2). Race and ethnicity also remained significantly associated with PTB in this model. When compared with White infants, the prevalences of PTB were higher for infants born to Black (PR, 1.28; 95% CI, 1.21-1.35), Hispanic (PR, 1.13; 95% CI, 1.08-1.18), and Asian or Pacific Islander birthing parents (PR, 1.15; 95% CI, 1.08-1.22). Notable associated covariates included birthing parent age of 45 years or older (PR, 1.53; 95% CI, 1.24-1.89) and the presence of any perinatal health conditions of the birthing parent (PR, 2.37; 95% CI, 2.27-2.48). The interaction term between race and ethnicity and COI added to the full model was not significant. Results of the model with interaction term are not included in Table 2.

## Discussion

This cross-sectional study examined whether a multidimensional measure of neighborhood child opportunity was associated with PTB. Consistent with previously published data from Massachusetts,^25^ we found inequities in PTB across racial and ethnic categories. Specifically, Black birthing parents had a higher risk of delivering preterm than all other racial and ethnic groups. We also found that more than half of the infants of Black and Hispanic birthing parents were born into very low opportunity neighborhoods compared with much smaller proportions of those born to White and Asian or Pacific Islander birthing parents. Furthermore, we identified a graded association between lower levels of neighborhood opportunity and increased prevalence of PTB even after adjusting for individual-level risk factors. This association has been documented with other child health outcomes, including mortality,^14^ hospitalization for ambulatory care sensitive conditions,^26^ and life expectancy at birth.^27^ Our findings also align with those of a small study in Albany, New York, which found that neighborhoods with a higher COI score were associated with higher birthweight, an outcome closely associated with gestational age.^28^ Our estimate of the magnitude of association of living in a very low opportunity neighborhood with PTB aligned with Ncube et al’s^5^ finding that residential disadvantage was associated with an approximately 25% increased risk of PTB overall.

We also investigated whether differential exposure to neighborhood opportunity by race and ethnicity accounted for any of the observed racial and ethnic inequities in PTB in 3 major metropolitan areas in Massachusetts. Associations between race and ethnicity and PTB were attenuated for Black and Hispanic groups by the inclusion of COI in a model, suggesting that COI might account for some of the racial inequity observed in a simple model. The inclusion of other covariates in a full model eliminated the significance of neither COI nor race and ethnicity, suggesting that these variables still had an independent association with PTB. In consideration of the 3 criteria for assessing racial disparities outline by Ward et al,^22^ our findings satisfy the criteria of a higher prevalence of the outcome (PTB) among Black as well as Hispanic groups and a much higher prevalence of the outcome of interest (living in a very low opportunity area) among these same groups. The lack of a significant interaction between race and ethnicity and PTB does not disqualify COI as a potential driver of inequities because the other 2 criteria were satisfied.

As has been documented elsewhere,^17,29^ social opportunity closely maps onto the geographic distribution of racial and ethnic populations in both Massachusetts and across the country. The current residential patterning by race and ethnicity is the legacy of discriminatory housing policies, including redlining, that were legal well into the latter half of the 20th century.^30^ The segregation of children and their families into levels of social and environmental opportunity by race and ethnicity is defined as a form of structural racism.^31^ As such, our finding of an association of neighborhood opportunity with PTB, coupled with the disproportionate distribution of this exposure by race and ethnicity, suggests that this modifiable indicator of structural racism contributes to and maintains racial and ethnic inequities in PTB.^31^

### Strengths and Limitations

Our study has a number of strengths. Most studies on neighborhood effects have focused on single indicators of social disadvantage, such as violence or income inequality.^14^ However, evidence points to the importance of using a more holistic measure of neighborhood conditions for understanding patterns of children’s health across geographic areas.^29^ By using a complex measure, our study points to many potential areas of community investment that could reduce racial and ethnic inequities in PTB. Additionally, our linkage of the COI to individual birth certificates allowed us to control for a variety of individual-level social and health characteristics associated with risk of PTB and estimate the unique association of opportunity with PTB risk.

This study also has several limitations that warrant mention. First, we conducted a complete-case analysis, excluding 3.8% of the observations from our original dataset that had any missing covariates. Although this was a small proportion, because the data were not missing at random, it is possible that our estimates were biased by these exclusions.^32^ Next, this analysis used version 1.0 of the COI, first developed in 2014. There are newer versions of the COI available; however, COI 1.0 contains geocoded data only for 2010, whereas COI 2.0 contains some data from 2015, which is after most births in this sample.^29^ In addition, although the COI provides a holistic view of the neighborhood, it captures neither length of residence nor changes in neighborhood conditions over time but only residence at the time of delivery. As such, the COI may not always accurately reflect cumulative exposure to opportunity level. However, because most people tend to stay in the same or similar opportunity area over time, it is likely robust as a measure of lifetime exposure.^32^ The generalizability of our findings is somewhat limited by COI 1.0 only covering births in the 3 large metropolitan areas of Massachusetts; however, our analytic dataset included nearly 80% of Massachusetts singleton births. Of note, because of the need for statistical power, categories of race and ethnicity contain considerable heterogeneity and possibly mask differences in opportunity within groups. Because our data were cross-sectional, it is possible that some covariates in our full model were mediators between COI and gestational age and, as such, inappropriately attenuated the COI estimates. Our measure of any perinatal health conditions depended on the birth certificate, which is known to be a less valid source of health data than administrative data.^33^ Lastly, Massachusetts leads the nation in both prevalence of coverage and proximity to advanced health care services.^34^ Because the COI is normed to local averages, ratings of low or very low health and environmental opportunity in Massachusetts would be considered moderate or even high in areas with lower overall access. As such, our findings should be interpreted with caution when considering regions where health care access is lower.

## Conclusions

The long-standing maldistribution of social opportunity on the basis of race and ethnicity or structural racism^26^ likely contributes to and perpetuates racial and ethnic gaps in PTB. Longitudinal studies are needed to establish the specific pathways between neighborhood of residence and health outcomes over time. Researchers should also investigate whether substantial and lasting investments in lower social opportunity areas, where Black and Hispanic or Latine children are more likely to be born and raised, reduce inequities in PTB and other adverse birth outcomes.

## Notes

Supplement 1. **eTable.** Comparing Distribution of Complete-Case Dataset (n = 267 553) to Original Dataset (n = 280 495)

Supplement 2.
Data Sharing Statement
