# Supplementary material for: Neighborhood Child Opportunity and Preterm Birth Rates by Race and Ethnicity
Source: JAMA Netw Open. 2024 Sep 11;7(9):e2432766. doi: 10.1001/jamanetworkopen.2024.32766 (PMC11391324; doi:10.1001/jamanetworkopen.2024.32766)
Supplement: Supplement 1. — eTable. Comparing Distribution of Complete-Case Dataset (n = 267 553) to Original Dataset (n = 280 495) [file jamanetwopen-e2432766-s001.pdf]

Supplemental Online Content

Belanoff C, Black A, Ncube C, Acevedo-Garcia D, Almeida J. Neighborhood child opportunity and preterm birth rates by race and ethnicity. *JAMA Netw Open*. 2024;7(9):e2432766. doi:10.1001/jamanetworkopen.2024.32766

eTable. Comparing Distribution of Complete-Case Dataset (n = 267 553) to Original Dataset (n = 280 495)

This supplemental material has been provided by the authors to give readers additional information about their work.

© 2024 Belanoff C et al. *JAMA Network Open*.

eTable 1. Comparing Distribution of Complete-Case Dataset (n = 267 553) to Original Dataset (n = 280 495)

	Complete-Case, Total n (%)	Original, Total n (%)
Race and ethnicity		
American Indian	n/a	824 (0.3)
Non-Hispanic Asian/Pacific Islander	26,948 (10.1)	27,619 (9.9)
Non-Hispanic Black	26,911 (10.1)	28,290 (10.1)
Hispanic	50,559 (18.9)	52,608 (18.8)
Non-Hispanic White	163,135 (61.0)	167,182 (59.6)
Non-Hispanic Other	n/a	1,502 (0.5)
Unknown	n/a	2,470 (0.9)
Child Opportunity Index Level		
Very High	50,333 (18.8)	52,535 (18.7)
High	49,030 (18.3)	51,134 (18.2)
Moderate	52,907 (19.8)	55,386 (19.8)
Low	49,004 (18.3)	51,476 (18.4)
Very Low	66,279 (24.8)	69,964 (24.9)
Metropolitan Area of Residence		
Boston	198,992 (74.4)	209,511 (74.7)
Springfield	30,954 (11.6)	31,856 (11.4)
Worcester	37,607 (14.1)	39,128 (14.0)
Age		
12 to 19	10,152 (3.8)	10,611 (3.8)
20 to 25	46,363 (17.3)	48,488 (17.3)
26 to 34	149,244 (55.8)	156,208 (55.7)
35 to 39	50,067 (18.7)	52,728 (18.8)
40 to 44	10,969 (4.1)	11,639 (4.2)
45+	758 (0.3)	821 (0.3)
Marital Status		
Divorced	5,659 (2.1)	5,944 (2.1)
Married	179,254 (67.0)	187,756 (66.9)
Never married	82,428 (30.8)	86,565 (30.9)
Other relationship	212 (0.1)	230 (0.1)
Nativity		
Unknown	n/a	18 (0.0)
US-Born	181,089 (67.7)	92,327 (32.9)
Non-US-Born	86,464 (32.3)	188,150 (67.1)
Education		
Less than High School	26,499 (9.9)	27,571 (9.8)
High School	45,050 (16.8)	46,737 (16.7)
Some College	65,720 (24.6)	67,722 (24.1)
4-Year Degree	67,848 (25.4)	69,839 (24.9)

© 2024 Belanoff C et al. *JAMA Network Open*.

Graduate School	62,436 (23.3)	64,090 (22.9)
Unknown	n/a	4,536 (1.6)
Private Insurance for Delivery		
Yes	206,709 (77.3)	215,747 (76.9)
No	60,844 (22.7)	64,748 (23.1)
Any Perinatal Health Conditions**		
No	111,895 (41.8)	117,183 (41.8)
Yes	155,658 (58.2)	163,294 (58.2)
Missing	n/a	18 (0.0)
Pre-pregnancy Body Mass Index (BMI)		
< 18.5	7,541 (2.8)	7,703 (2.8)
18.5 to 24.9	132,738 (49.6)	135,609 (48.4)
25.0 to 29.9	72,866 (27.2)	74,538 (26.6)
30 to <35	30,764 (11.5)	31,544 (11.3)
35 to <40	14,253 (5.3)	14,573 (5.2)
40+	9,391 (3.5)	9,626 (3.4)
Unknown	n/a	6,902 (2.5)

© 2024 Belanoff C et al. *JAMA Network Open*.
