# Supplementary material for: Neighborhood Child Opportunity and Preterm Birth Rates by Race and Ethnicity
Source: JAMA Netw Open. 2024 Sep 11;7(9):e2432766. doi: 10.1001/jamanetworkopen.2024.32766 (PMC11391324; doi:10.1001/jamanetworkopen.2024.32766)
Supplement: Supplement 2. — Data Sharing Statement [file jamanetwopen-e2432766-s002.pdf]

Data Sharing Statement

Belanoff. Neighborhood Child Opportunity and Preterm Birth Rates by Race and Ethnicity in Massachusetts, 2011-2015. *JAMA Netw Open*. Published September 11, 2024. doi:10.1001/jamanetworkopen.2024.32766

Data
Data available: No

Additional Information
Explanation for why data not available: The authors are not authorized to share individual level birth file data. The data used from the Child Opportunity Index are publicly available at DiversityDataKids.org.
